# Identifying vaccination deserts: The availability and distribution of pharmacists with authorization to administer injections in Ontario

**DOI:** 10.1177/17151635221115183

**Published:** 2022-08-05

**Authors:** Sherilyn K.D. Houle, Patrick Timony, Nancy M. Waite, Alain Gauthier

## Abstract

**Introduction:**

Pharmacist-administered immunizations have been associated with improved vaccination rates; however, little is known about whether areas with little to no access to this service (“vaccination deserts”) exist. The objective of this work is to determine the geographic availability of pharmacists with authorization to administer injections in the province of Ontario.

**Methods:**

Ontario College of Pharmacists registry data were used to identify patient care–providing pharmacists in community pharmacies and their ability to administer injections. Their number of hours worked was converted into full-time equivalents (FTEs), assuming 40 hours per week represents 1 FTE. Practice site(s) were mapped by postal code and presented by Public Health Unit (PHU) area. Communities within PHUs were further categorized as urban or rural and northern or southern, with ratios of FTEs per 1000 population calculated for both injection-trained and non-injection-trained pharmacists.

**Results:**

In total, 74.6% of Ontario’s practising community pharmacists are authorized to provide injections. Northern PHUs had slightly better access to pharmacist injectors (0.61 FTEs/1000 overall vs 0.56/1000 in the south), while rural communities had lower availability (0.41 FTEs/1000) than urban communities (0.58 FTEs/1000). PHUs with greater population size and density had greater availability of pharmacist immunizers, while PHUs with greater land area were more likely to not have any immunizing pharmacists present (*p* < 0.001 for all).

**Discussion:**

As pharmacists increasingly become preferred vaccination providers, awareness of disparities related to access to pharmacy-based immunizations and collaboration with public health and primary care providers to address them (e.g., through mobile vaccination clinics) will be required to ensure equitable access. *Can Pharm J (Ott)* 2022;155:xx-xx.

**Knowledge into Practice**

- Geographic disparities exist related to access to primary care services; however, little is known about whether this extends to pharmacy-provided vaccination services.
- While three-quarters of Ontario pharmacists are authorized to administer injections, the ability of Ontarians to access these pharmacists varies significantly, depending on where in the province a person resides.
- With pharmacists increasingly becoming preferred vaccination providers, novel approaches may be needed to reach populations with limited access to a pharmacy or to provide training opportunities for pharmacists practising in more remote communities who are not currently authorized to administer injections.

## Introduction

Vaccinations are a cornerstone of public health, with vaccine hesitancy identified as one of the leading threats to health worldwide.^1^ Pharmacists in Washington were the first to be formally trained in the administration of injections in 1994^2^—this expansion to the scope of practice of pharmacists is now present in 11 Canadian provinces and territories.^3^

Pharmacy-based vaccination services have resulted in modest but significant increases in overall vaccination rates, often attributed to pharmacists’ accessibility both geographically and regarding hours of operation.^4-6^ Research has found that pharmacies successfully reach working-age adults for influenza vaccinations,^7^ with 1 study reporting that 30% of vaccinations were administered by a pharmacy chain outside of usual operating hours of physician offices and public health clinics.^8^

The availability of primary care providers has been found to disproportionately benefit those who live in urban centres, across both pharmacists and physicians. For example, while a study found that 92.9% of the Ontario population overall was attached to a regular primary care physician, this was reduced to 86.9% and 86.7% in the northeast and northwest regions of the province, respectively, which tend to have fewer high-population centres.^9^ Additionally, the ability for an individual to see their primary care physician the same or next day has also been found to differ by geographic region, with 53% of Ontarians residing in the Greater Toronto Area being able to do so vs 24% of northern Ontarians.^10^ When considering the availability of pharmacy-based primary care services specifically, a study by Law et al.^11^ found that while 90.7% of Ontarians live within 5 km of a pharmacy, this is true for only 40.9% of rural residents.

Regarding the availability of vaccination services at community pharmacies, unlike physicians and nurses, the scope of practice for pharmacists to administer injections is not universal upon licensure. In Canada, pharmacists wishing to perform this service must complete a vaccination and injection training program approved by each jurisdiction’s pharmacy regulatory body,^12^ with this authorization added to their licensure status upon successful completion. As this licensure information is publicly available, and pharmacists must report to their licensing bodies the pharmacies at which they practise, it is possible to identify a geographic area’s level of pharmacist vaccinator coverage or, conversely, identify “vaccination deserts” where this service is not readily available to the public. The objective of this work is to determine the geographic availability of pharmacists with authorization to administer injections in the province of Ontario.

**Mise En Pratique Des Connaissances**

- Il existe des disparités géographiques liées à l’accès aux services de soins primaires; toutefois, on ne sait pas si cela s’étend aux services de vaccination fournis par les pharmacies.
- Bien que les trois quarts des pharmaciens de l’Ontario soient autorisés à administrer les injections, la capacité des Ontariens à y accéder varie considérablement selon l’endroit où ils habitent dans la province.
- Étant donné que les pharmaciens deviennent de plus en plus des fournisseurs de vaccination privilégiés, de nouvelles approches peuvent être nécessaires pour atteindre les populations ayant un accès limité à une pharmacie ou pour offrir des occasions de formation aux pharmaciens exerçant dans des communautés plus éloignées qui ne sont pas actuellement autorisées à administrer des injections.

## Methods

### Data source and inclusion criteria

Registry data from the Ontario College of Pharmacists (OCP) were accessed for this secondary analysis of pharmacists licensed to provide patient care (Part A licensure) in Ontario. Pharmacists were included in the analysis if they 1) reported an active practice site as of December 20, 2018; 2) specified the number of hours worked at each site (sites with 0 hours per week reported or missing data were excluded); and 3) reported working in a community pharmacy setting. Practice in hospitals and nonaccredited practice sites were excluded, as the intention of our analysis was to assess the availability of pharmacist vaccination services to the general public.

### Injection-trained pharmacist availability

Pharmacist availability was calculated by converting the hours worked at each practice site into full-time equivalents (FTEs), assuming a 40-hour work week as 1 FTE. Since the OCP registry collects hours worked as 1 to 14 hours, 15 to 29 hours, 30 to 39 hours, and 40 hours or more, the midpoint of each range was selected to represent the number of hours worked at each site, which was then converted to FTEs. Thus, 1 to14 hours was converted to 7 hours or 0.175 FTEs, 15 to 29 hours was converted to 22 hours or 0.55 FTEs, 30 to 39 hours was converted to 35 hours or 0.875 FTEs, and 40 hours or more was estimated to represent 40 hours or 1 FTE. Although the midpoint of each range may not be an accurate representation of hours worked, it is reasonable to assume that this would provide a close approximation when averaged over the entire pharmacist population. Pharmacists who reported having been trained to administer injections were assumed to be providing this service and were compared to the population distribution using the 2016 Canadian Census.^13^

### Geographic definitions

Census geographies were attributed to each practice site named in the OCP registry using the 2018 Postal Code Conversion File (PCCF) from Statistics Canada. First, Census Subdivisions (CSDs) or municipality were identified from the practice addresses reported on the registry. CSDs are used in this study to delineate communities. Next, Statistics Canada’s Statistical Area Classifications (SACs), which are attributed to CSDs, were used to differentiate urban communities (i.e., CSDs classified as Census Metropolitan Areas or Census Agglomerations) from rural communities (i.e., all remaining CSDs). Communities were then grouped by Public Health Units (PHUs) using Statistics Canada’s health region-to-census geography correspondence file.^14^ Communities were considered northern if they were located within the following PHUs: North Bay Parry Sound District Health Unit, Timiskaming Health Unit, Sudbury and District Health Unit, Porcupine Health Unit, The District of Algoma Health Unit, Thunder Bay District Health Unit and Northwest Health Unit.

In Ontario, local PHUs are responsible for the implementation of vaccination programs as well as the surveillance of vaccination coverage.^15^ Thus, results are provided at the local PHU level to aid in the effective planning of vaccination efforts. Data on population size, population density (per square kilometre), and land area (in square kilometres) of PHUs were accessed from Statistics Canada’s 2016 census profiles.^16^ Results are also aggregated by large geographic regions (i.e., north vs south and rural vs urban) to provide a general consideration of the distribution of injection-trained pharmacists. Finally, to compare pharmacist availability in PHUs of varying sizes, ratios of pharmacist FTEs per 1000 residents were calculated.

### Statistical analysis

To practise pharmacy in Ontario, pharmacists must be registered with the OCP; therefore, as data contained in the OCP registry include the entire pharmacist population, inferential statistics are not necessary to analyze these data. A series of descriptive and nonparametric analyses was used to compare injection-trained to non-injection-trained pharmacists and to explore the distribution of these among local PHUs. Pearson and Spearman correlations were used to explore the relationship between PHU characteristics (population size, population density and land area) and pharmacist availability.

### Ethics

Research ethics approval was granted by the University of Waterloo and Laurentian University. Data-sharing agreements were struck between the OCP and the University of Waterloo, as well as the University of Waterloo and Laurentian University, allowing confidential data transfer between OCP and the appropriate researchers.

## Results

A total of 11,436 pharmacists with an active practice in community pharmacy were included in the analyses (Table 1). Nearly three-quarters (*n* = 8530, 74.6%) were authorized to provide injections. Injection-trained pharmacists were significantly more likely to be women (55.1%) than non-injection-trained pharmacists (50.1%) (*p* < 0.001). While nearly all pharmacists in Ontario reported being able to practise in English, injection-trained pharmacists were significantly less likely to report being able to practise in French (6.8% vs 8.2% of non-injection-trained pharmacists, *p* < 0.05) and slightly less likely to be able to practise in another language (51.2% vs 52.0% of non-injection-trained pharmacists, *p* = 0.457). Injection-trained pharmacists had been in practice for fewer years, with 60% having graduated within the last 20 years, compared to 40% of non-injection-trained pharmacists. A nonparametric Mann-Whitney *U* test confirmed that non-injection-trained pharmacists had been in practice for significantly longer (mean = 25 years) than injection-trained pharmacists (mean = 18 years) (*p* < 0.001). Injection-trained pharmacists were more likely than non-injection-trained pharmacists to have been educated in Canada (47% vs 44.5%; *p* < 0.05), whereas non-injection-trained pharmacists were more likely to have been trained in the United States (8.6% vs 5.8% of injection-trained pharmacists; *p* < 0.001). Finally, injection-trained pharmacists were more likely to practise at a single practice site (64.2%) than their non-injection-trained counterparts (59.8%), with a Mann-Whitney *U* test confirming that non-injection-trained pharmacists practised at significantly more sites (*p* < 0.001).

**Table 1:** Characteristics of actively practising community pharmacists in Ontario

	Injection-trained			Non-injection-trained			Total pharmacists		
	*n*	Within group %	Between group %	*n*	Within group %	Between group %	*n*	Within group %	Between group %
Total	8530	100.0	74.6	2906	100.0	25.4	11,436	100.0	100.0
**Gender**									
Female	4699	55.1	76.3	1457	50.1	23.7	6156	53.8	100.0
Male	3831	44.9	72.6	1449	49.9	27.4	5280	46.2	100.0
**Language of competence**									
English	8529	100.0	74.6	2902	99.9	25.4	11,431	100.0	100.0
French	584	6.8	71.0	239	8.2	29.0	823	7.2	100.0
Other	4370	51.2	74.3	1512	52.0	25.7	5882	51.4	100.0
**Years since graduation**									
<1	122	1.4	89.7	14	0.5	10.3	136	1.2	100.0
1-5	1365	16.0	87.1	203	7.0	12.9	1568	13.7	100.0
6-10	1421	16.7	82.1	310	10.7	17.9	1731	15.1	100.0
11-20	2229	26.1	77.3	653	22.5	22.7	2882	25.2	100.0
21-30	1833	21.5	73.7	655	22.5	26.3	2488	21.8	100.0
31-40	1193	14.0	65.3	635	21.9	34.7	1828	16.0	100.0
41-50	339	4.0	48.4	362	12.5	51.6	701	6.1	100.0
51+	28	0.3	27.5	74	2.5	72.5	102	0.9	100.0
**Country of education**									
Canada	4010	47.0	75.6	1293	44.5	24.4	5303	46.4	100.0
United States	496	5.8	66.5	250	8.6	33.5	746	6.5	100.0
Other	4024	47.2	74.7	1363	46.9	25.3	5387	47.1	100.0
**Number of practice sites**									
1	5480	64.2	75.9	1739	59.8	24.1	7219	63.1	100.0
2	2025	23.7	73.0	748	25.7	27.0	2773	24.2	100.0
3-5	947	11.1	72.1	367	12.6	27.9	1314	11.5	100.0
6-10	61	0.7	64.9	33	1.1	35.1	94	0.8	100.0
11-20	11	0.1	39.3	17	0.6	60.7	28	0.2	100.0
20+	6	0.1	75.0	2	0.1	25.0	8	0.1	100.0

### Geographic distribution

**Figure 1:**
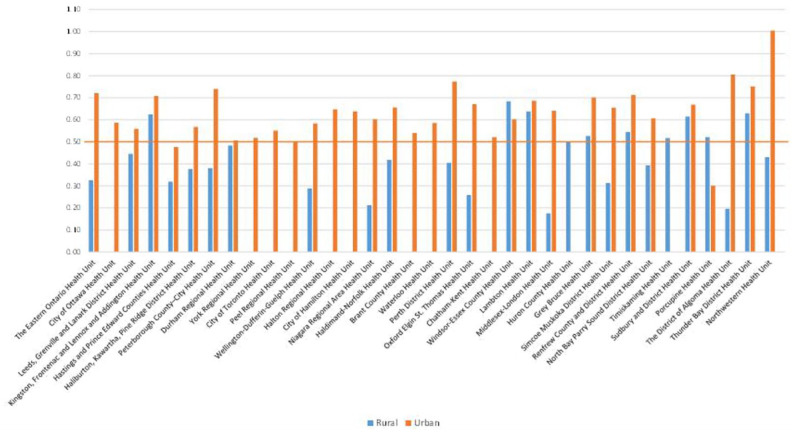
Injection-trained pharmacist availability (in FTEs/1000 population), by health unit

The distribution of injection-trained pharmacists throughout each of Ontario’s PHUs and between rural and urban areas is found in Appendix 1. Overall, the pharmacist FTE to 1000 population ratio in Ontario was 0.56. A “threshold” ratio of 0.5 FTEs per 1000 population was therefore used to guide our analysis. Although only 6.2% of injection-trained pharmacist FTEs practised in northern PHUs, these northern health units had slightly better access, with a ratio of 0.61 FTEs per 1000 population in the north compared to 0.56 in the south. Generally speaking, rural communities had lower availability of injection-trained pharmacists (0.41 FTEs per 1000 population) than urban communities (0.58). However, much variability was observed both between and within PHUs (Figure 1). Overall, all but 3 PHUs had above-threshold availability of injection-trained pharmacists, with ratios ranging from a high of 0.74 FTEs per 1000 population in the Thunder Bay District Health Unit to a low of 0.41 FTEs per 1000 population in the Porcupine Health Unit. Within PHUs, there was a clear trend for greater availability of injection-trained pharmacists in urban communities, with only 2 PHUs having below-threshold availability in their urban areas, namely, the Hastings and Prince Edward Counties Health Unit (0.48 FTEs per 1000 population) and the Porcupine Health Unit (0.30 FTEs per 1000 population). Rural communities were much more likely to experience below-threshold availability, with rural areas in 16 out of 26 PHUs (5 PHUs did not contain rural communities) falling below the 0.5 FTE per 1000 population threshold.

**Figure 2:**
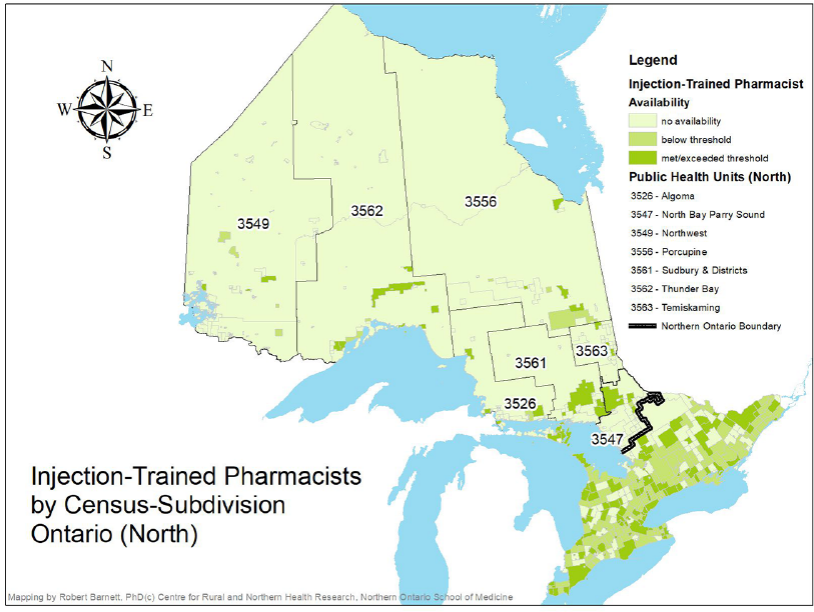
Injection-trained pharmacist availability, northern Ontario

**Figure 3:**
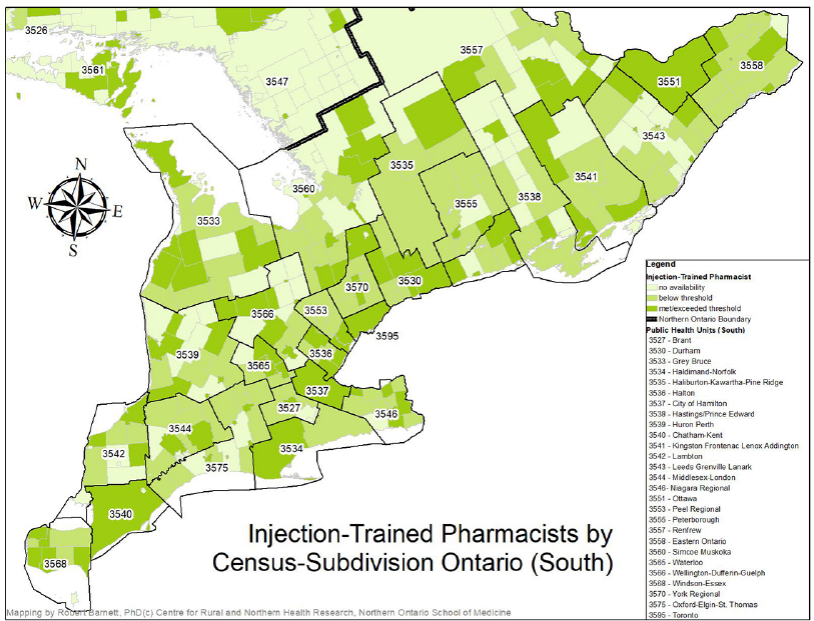
Injection-trained pharmacist availability, southern Ontario

Although at a macro level, almost all PHUs met or exceeded the threshold of availability of injection-trained pharmacists, and northern PHUs had slightly better access than southern PHUs, local realities within PHUs reveal access concerns that could challenge local immunization efforts. Appendix 2 contains a breakdown by PHU of the number of communities that fall under each of the following availability conditions: no availability of injection-trained pharmacists (0 FTEs per 1000 population), below-threshold availability (≤0.49 FTEs per 1000 population) or met/exceeded the threshold (≥0.5 FTEs per 1000 population). This information is also presented graphically in Figures 2 and 3.

As over 25% of pharmacists in Ontario are not authorized to provide injections, these non-injection-trained pharmacists represent potential capacity if they pursue injection authorization. Thus, Appendix 2 includes a combination of injection and non-injection-trained pharmacists to determine whether training all Ontario community pharmacists would improve potential availability based on the 0.5 FTE/1000 population threshold of injecting pharmacists. Table 2 provides a summary of the results from Appendix 2 aggregated by large geographic regions (i.e., urban south, rural south, urban north, rural north); however, we encourage readers to review Appendix 2 for a better appreciation of local PHU realities.

**Table 2:** Impact of authorizing all pharmacists to administer injections by count of Census Subdivisions and geographic region

Injection authorized	No availability, *n* (%)			Below-average availability, *n* (%)		
Combination injection authorized and nonauthorized	Still no availability	Below-average availability	Average/above-average availability	Below-average availability	Average/above-average availability	Above-average availability, *n* (%)
Urban south	17 (13)			44 (35)		66 (52)
	17 (13)	0	0	28 (22)	16 (13)	
Rural south	66 (38)			56 (33)		50 (29)
	61 (35)	5 (3)	0	41 (24)	15 (9)	
Urban north	17 (59)			4 (14)		8 (28)
	16 (55)	1 (3)	0	4 (14)	0	
Urban south	205 (83)			11 (4)		31 (13)
	199 (81)	1 (0.4)	5 (2)	4 (2)	7 (3)	

Despite southern PHUs as a whole having a slightly smaller ratio of injection-trained pharmacist FTEs per 1000 population than northern PHUs, southern PHUs also had fewer communities with no injection-trained pharmacist availability. Only 13% of communities in the urban south and 38% of communities in the rural south had no injection-trained FTEs compared to 59% of communities in the urban north and 83% of communities in the rural north. Furthermore, urban and rural communities in the south were more likely to have met or exceeded the availability threshold (52% and 29%, respectively) than urban and rural communities in the north (28% and 13%, respectively). The addition of non-injection-trained pharmacists had limited impact on availability, with only 7 communities moving from no availability to below-threshold availability (5 in the rural south, 1 in the urban north and 1 in the rural north) and 38 communities moving from below-threshold availability to having met or exceeded threshold availability (16 in the urban south, 15 in the rural south and 7 in the rural north).

Besides differences in the availability of injection-trained pharmacists, PHUs will also need to contend with other local specificities, which can impede vaccination efforts, namely, the size of the area and of the populations they serve. A series of Pearson and Spearman correlations was used to explore the relationship between these PHU characteristics and the availability of injection-trained pharmacists. First, there was a very strong positive and statistically significant correlation (Pearson *r* = 0.99, *p*(one-tailed) < 0.001) between population size and injection-trained pharmacist FTEs, such that PHUs with larger populations also had more injection-trained pharmacist FTEs. There was also a strong positive and statistically significant correlation (Pearson *r* = 0.93, *p*(one-tailed)< 0.001) between population density (defined as the number of residents per square kilometre) and the number of injection-trained FTEs, such that the availability of these pharmacists became greater as the population density of the PHUs also became greater. Finally, there was a slightly weaker yet still positive and statistically significant correlation (Spearman *r* = 0.85, *p*(one-tailed) < 0.001) between PHU land areas (in square kilometres) and the number of communities with no injection-trained pharmacists—as PHUs became larger in size, they also had more communities with no injection-trained pharmacists available.

## Discussion

With nearly three-quarters of Ontario community pharmacists authorized to administer injections, as well as millions of influenza and COVID-19 vaccines administered by pharmacists this past year alone,^17,18^ the uptake of this service by both pharmacists and patients has been greater than many other full-scope activities in the province.^19-21^ However, this analysis demonstrates that there are regions where the public may not be able to readily access this important service.

In general, northern regions had greater availability than southern regions, with rural communities having less access than urban communities within the same PHU. However, despite greater pharmacist injector FTE availability per capita in northern Ontario, 3 strong and significant correlations were observed that suggest accessibility issues may exist for those living in some northern regions. Specifically, increased overall population and population density per square kilometre were associated with greater availability, and PHUs covering a larger land area were more likely to have communities with no pharmacist injector availability. Therefore, residents of rural areas within geographically large northern PHUs may experience significant difficulty accessing pharmacist vaccination services given the lack of local vaccination capacity, their distance to neighbouring pharmacies and seasonal travel conditions. Such challenges are also well documented within the rural health care field of research.^22^ As pharmacists become increasingly relied-upon providers of vaccinations and, in some cases, substituting for services traditionally provided by public health clinics or medical offices, it is important to be aware of the potential for vaccination deserts and to devise novel strategies to reach these populations. Interprofessional and interorganizational collaboration and innovations such as mobile vaccination clinics will be required to ensure more equitable access. For example, research on the acceptability of a pharmacy student-run mobile influenza vaccination clinic reported high patient satisfaction with this model, with convenience cited as the primary reason for receiving this service by 92% of survey respondents visiting the clinic.^23^

Differences in access to other primary care services have also been identified between urban and rural regions in Canada, with lower accessibility for residents of rural communities, consistent with our findings.^24-26^ Most related to our work, research by Sibley and Weiner^24^ reported that the lowest influenza vaccination rates were observed among the most rural Canadians, and a recent report by Environics Analytics found higher rates of COVID-19 vaccination hesitancy among rural populations.^27^ Furthermore, as indicated in Table 2 and Appendix 2, our analysis has found that a number of communities do not have access to pharmacists in their Census Subdivision at all, as evidenced by communities that would continue to have no or below-threshold availability even if all pharmacists practising in that region were authorized to administer injections. It may be hypothesized that communities without access to a pharmacy may also lack local access to other primary care providers, indeed representing true vaccination deserts.

Other research in North America and Europe has reported on the existence of “medication deserts,” where the availability of commonly prescribed medications may vary based on the socioeconomic status of the community in which they are located.^28^ Taken together, evidence suggests that where a person lives may affect their ability to access necessary medical services—a care gap that policymakers and health care professionals should make efforts to resolve. Future research should also consider the availability of linguistically concordant vaccination services, as our data have identified that injection-trained pharmacists are less likely to be able to practise in French and that injection deserts are far more common in northern Ontario, where many Francophone populations reside, particularly in the northeast.^29^ A recent analysis of the distribution of French-speaking pharmacists using the same OCP data found a lower proportion of pharmacists who self-declare French as a language of competence in communities with larger Francophone populations.^30^ The inability to communicate in one’s preferred language may further affect vaccination uptake and hesitancy.^31^

### Limitations

As participation in influenza, COVID-19 and other vaccination programs is optional for pharmacists, limitations with the data may include an overestimation of the availability of pharmacist-provided vaccination services, as availability of the service was estimated using licensure data on those pharmacists indicating that they are currently authorized to administer injections in Ontario. Our analysis therefore assumed that all pharmacists with the potential to administer vaccines did indeed do so. We also excluded pharmacy students and registered pharmacy technicians from the analysis, which may represent an underestimation of FTE availability of vaccinators through community pharmacies; however, we expect there to be no change to the number of communities with no access to pharmacy-delivered vaccination services, as these individuals must be working under the supervision of a licensed pharmacist. Additionally, as both pharmacy programs in Ontario offer injection and vaccination training to students, and our analysis found that pharmacists with fewer years of experience were more likely to be authorized to administer injections than more experienced pharmacists, we may expect the proportion of practising pharmacists with authorization to administer injections to increase with time. In fact, our data show that nearly 90% of pharmacists who had graduated within the past 5 years were authorized to administer injections. Finally, while current legislation permits registered pharmacy technicians in Ontario with recognized training to administer the COVID-19 and influenza vaccines,^32^ future efforts to expand this to include other vaccinations is expected to further improve pharmacy-based vaccination service availability for the public. With over 5400 pharmacy technicians licensed in Ontario,^33^ this could represent a significant addition to the vaccinator workforce.

## Conclusion

These analyses demonstrate that despite the fact that nearly three-quarters of Ontario’s community pharmacists are authorized to administer vaccinations, public access to this important service is a function of where an individual lives. Considerations such as northern vs southern Ontario residence, urban or rural community characteristic, population density, and geographic area of one’s Public Health Unit can influence the true accessibility of this service. While telepharmacy and other virtual care options may suffice for many other medication management needs, reaching individuals living in vaccination deserts will likely require interprofessional and interorganizational collaboration to coordinate mobile vaccination clinics in more remote communities. As pharmacies become increasingly used venues for vaccination efforts, awareness of those communities without a local community pharmacy and creative approaches to provide care to such communities will be required to ensure equitable access to this important public health service. ■

## Supplemental Material

sj-pdf-1-cph-10.1177_17151635221115183 – Supplemental material for Identifying vaccination deserts: The availability and distribution of pharmacists with authorization to administer injections in Ontario Click here for additional data file. Supplemental material, sj-pdf-1-cph-10.1177_17151635221115183 for Identifying vaccination deserts: The availability and distribution of pharmacists with authorization to administer injections in Ontario by Sherilyn K.D. Houle, Patrick Timony, Nancy M. Waite and Alain Gauthier in Canadian Pharmacists Journal / Revue des Pharmaciens du Canada

sj-pdf-2-cph-10.1177_17151635221115183 – Supplemental material for Identifying vaccination deserts: The availability and distribution of pharmacists with authorization to administer injections in Ontario Click here for additional data file. Supplemental material, sj-pdf-2-cph-10.1177_17151635221115183 for Identifying vaccination deserts: The availability and distribution of pharmacists with authorization to administer injections in Ontario by Sherilyn K.D. Houle, Patrick Timony, Nancy M. Waite and Alain Gauthier in Canadian Pharmacists Journal / Revue des Pharmaciens du Canada

## References

[1] World Health Organization. Ten threats to global health in 2019. Available: https://www.who.int/news-room/spotlight/ten-threats-to-global-health-in-2019 (accessed May 25, 2021).

[2] Hogue MD Grabenstein JD Foster SL Rothholz MC. Pharmacist involvement with immunizations: a decade of professional advancement. J Am Pharm Assoc 2006;46(2):168-79. 10.1331/154434506776180621 16602227

[3] Canadian Pharmacists Association. Pharmacists’ Expanded Scope of Practice. Available: https://www.pharmacists.ca/pharmacy-in-canada/scope-of-practice-canada/ (accessed May 25, 2021).

[4] Isenor JE Edwards NT Slayter KL , et al. Impact of pharmacists as immunizers on vaccination rates: a systematic review and meta-analysis. Vaccine 2016;34(47):5708-23. 10.1016/j.vaccine.2016.08.085 27765379

[5] Baroy J Chung D Frisch R Apgar D Slack MK. The impact of pharmacist immunization programs on adult immunization rates: a systematic review and meta-analysis. J Am Pharm Assoc 2016;56(4):418-26. 10.1016/j.japh.2016.03.006 27450138

[6] Buchan SA Rosella L Finkelstein M , et al. Impact of pharmacist administration of influenza vaccines on uptake in Canada. Can Med Assoc J 2017;189(4):e146-52. 10.1503/cmaj.151027 PMC5266568 27503864

[7] Waite NM Cadarette SM Campitelli MA , et al. Characteristics of patients vaccinated against influenza in physician offices versus pharmacies and predictors of vaccination location: a cross-sectional study. CMAJ Open 2019;7(2):E421-9. 10.9778/cmajo.20180189 PMC6588543 31227484

[8] Goad JA Taitel MS Fensterheim LE Cannon AE. Vaccinations administered during off-clinic hours at a national community pharmacy: implications for increasing patient access and convenience. Ann Fam Med 2013;11(5):429-36. 10.1370/afm.1542 PMC3767711 24019274

[9] Hay C Pacey M Bains N. Understanding the unattached population in Ontario: evidence from the Primary Care Access Survey (PCAS). Healthc Policy 2010;6(2):33-47. 22043222 PMC3016634

[10] Health Quality Ontario. Measuring Up 2016: A yearly report on how Ontario’s health system is performing. Available: https://www.hqontario.ca/portals/0/Documents/pr/measuring-up-2016-en.pdf (accessed May 30, 2021).

[11] Law MR Dijkstra A Douillard JA Morgan SG. Geographic accessibility of community pharmacies in Ontario. Healthc Policy 2011;6(3):36-45. 22294990 10.12927/hcpol.2011.22097 PMC3082386

[12] Houle SKD . Canadian pharmacists as immunizers: addressing questions related to this new scope of practice. Can J Public Health 2017;108(4):e418-20. 10.17269/CJPH.108.6119 PMC6972044 29120315

[13] Statistics Canada. Census of Population, Statistics Canada Catalogue no. 98-400-X2016348. Available: https://www12.statcan.gc.ca/census-recensement/2016/dp-pd/dt-td/Rp-eng.cfm?LANG=E&APATH=3&DETAIL=0&DIM=0&FL=A&FREE=0&GC=0&GID=0&GK=0&GRP=1&PID=110461&PRID=10&PTYPE=109445&S=0&SHOWALL=0&SUB=888&Temporal=2016,2017&THEME=118&VID=0&VNAMEE=&VNAMEF= (accessed May 30, 2021).

[14] Statistics Canada. Health regions: boundaries and correspondence with census geography. Available: https://www150.statcan.gc.ca/n1/pub/82-402-x/2017001/corr-eng.htm (accessed May 30, 2021).

[15] Government of Ontario. Ontario’s publicly funded immunization system: building on today’s strengths, innovating for the future. Available: https://www.health.gov.on.ca/en/common/ministry/publications/reports/immunization/docs/immun_sys_review_march2014_en.pdf (accessed May 30, 2021).

[16] Statistics Canada. Census profile, 2016 census. Available: https://www12.statcan.gc.ca/census-recensement/2016/dp-pd/prof/details/page.cfm?Lang=E&Geo1=HR&Code1=3595&Geo2=PR&Code2=35&SearchText=City%20of%20Toronto%20Health%20Unit&SearchType=Begins&SearchPR=01&B1=All&GeoLevel=PR&GeoCode=3595&TABID=1&type=0 (accessed May 30, 2021).

[17] Statistics Canada. Vaccine uptake in Canadian adults 2019. Available: https://www.canada.ca/en/public-health/services/publications/healthy-living/2018-2019-influenza-flu-vaccine-coverage-survey-results.html (accessed June 16, 2021).

[18] Cision. Alberta’s pharmacists administer 1 million COVID-19 shots. Available: https://www.newswire.ca/news-releases/alberta-s-pharmacists-administer-1-million-covid-19-shots-877691681.html (accessed June 16, 2021).

[19] Dolovich L Consiglio G MacKeigan L , et al. Uptake of the MedsCheck annual medication review service in Ontario community pharmacies between 2007 and 2013. Can Pharm J 2016;149(5):293-302. 10.1177/1715163516662670 PMC5032934 27708675

[20] Wong L Burden AM Liu YY , et al. Initial uptake of the Ontario Pharmacy Smoking Cessation Program: descriptive analysis over 2 years. Can Pharm J 2014;148(1):29-40. 10.1177/1715163514562038 PMC4294811 26759563

[21] Houle SKD Kozlovsky K Fernandes HVJ Rosenberg-Yunger Z . Uptake of travel health services by community pharmacies and patients following pharmacist immunization scope expansion in Ontario, Canada. Pharmacy 2019;7:35. 10.3390/pharmacy7020035 PMC6630201 31013879

[22] Strasser R. Rural health around the world: challenges and solutions. Fam Pract 2003;20(4):457-63. 10.1093/fampra/cmg422 12876121

[23] Hannings AN Duke LJ Logan LD , et al. Patient perceptions of student pharmacist-run mobile influenza vaccination clinics. J Am Pharm Assoc 2019;59(2):228-31. 10.1016/j.japh.2018.10.018 30578128

[24] Sibley LM Weiner JP. An evaluation of access to health care services along the rural-urban continuum in Canada. BMC Health Serv Res 2011;11:20. 21281470 10.1186/1472-6963-11-20 PMC3045284

[25] Shah T Milosavljevic S Bath B. Geographic availability to optometry services across Canada: mapping distribution, need and self-reported use. BMC Health Serv Res 2020;20:639. 32650762 10.1186/s12913-020-05499-6 PMC7350740

[26] Shah T Clark AF Seabrook JA Sibbald S Gilliland JA. Geographic accessibility to primary care providers: comparing rural and urban areas in Southwestern Ontario. Can Geogr 2020;64(1):65-78.

[27] Environics Analytics. VaccineInsights: where do we need to build confidence in vaccines? A data and analytics approach. Available: https://storymaps.arcgis.com/stories/7aceed148261444d8cb9c909f1ef1969 (accessed Aug. 11, 2021).

[28] Amstislavski P Matthews A Sheffield S , et al. Medication deserts: survey of neighborhood disparities in availability of prescription medications. Int J Health Geogr 2012;11:48. 23137192 10.1186/1476-072X-11-48 PMC3517332

[29] Timony PE Gauthier AP Hogenbirk JC Wenghofer EF. Promising quantities, disappointing distribution: investigating the presence of French-speaking physicians in Ontario’s rural Francophone communities. Rural Remote Health 2013;13(4):1-11. 24380635

[30] Timony PE Waite NM Houle SKD Violette R Gauthier AP . Le Pharmacien est disponible . . . (The Pharmacist is In): the availability and distribution of French-speaking pharmacists in Ontario. Minorités linguistiques et société/Linguistic Minorities and Society; 2022;18:175-196.

[31] Dubé E MacDonald NE. Vaccine acceptance: barriers, perceived risks, benefits, and irrational beliefs. In: Bloom BR Lambert P-H , eds. The vaccine book. New York: Academic Press; 2016. p. 507-28.

[32] Ontario College of Pharmacists. COVID-19: Information for Pharmacy Professionals. Available: https://www.ocpinfo.com/regulations-standards/novel-coronavirus-covid-19-professionals/ (accessed June 29, 2021).

[33] Ontario College of Pharmacists. Navigating change and putting patients first: 2021 annual report. Available: https://www.ocpinfo.com/wp-content/uploads/2022/04/ocp_annual_report_2021.pdf (accessed June 24, 2022).

