# Supplementary material for: Identifying vaccination deserts: The availability and distribution of pharmacists with authorization to administer injections in Ontario
Source: Can Pharm J (Ott). 2022 Aug 5;155(5):258–66. doi: 10.1177/17151635221115183 (PMC9445507; doi:10.1177/17151635221115183)
Supplement: sj-pdf-1-cph-10.1177_17151635221115183 – Supplemental material for Identifying vaccination deserts: The availability and distribution of pharmacists with authorization to administer injections in Ontario [file sj-pdf-1-cph-10.1177_17151635221115183.pdf]

APPENDIX 1 Injection trained pharmacist availability by Public Health Unit and rural or urban community size

	Total Population	Size (km^2)	FTEs per 1000 population	Rural		Urban	
				Population (n, % of total)	FTEs per 1000 population	Population (n, % of total)	FTEs per 1000 population
The Eastern Ontario Health Unit	200,036	5314	0.53	90,756 (45.4%)	0.33	109,280 (54.6%)	0.72
City of Ottawa Health Unit	923,370	2790	0.58	---	---	923,370 (100%)	0.59
Leeds, Grenville and Lanark District Health Unit	166,835	6419	0.50	81,795 (49.0%)	0.45	85,040 (51.0%)	0.56
Kingston, Frontenac and Lennox and Addington Health Unit	188,865	6627	0.68	31,680 (16.8%)	0.62	157,185 (83.2%)	0.71
Hastings and Prince Edward Counties Health Unit	159,095	7154	0.42	56,930 (35.8%)	0.32	102,165 (64.2%)	0.48
Haliburton, Kawartha, Pine Ridge District Health Unit	176,345	9066	0.49	66,570 (37.7%)	0.38	109,775 (62.3%)	0.57
Peterborough County-City Health Unit	136,000	3848	0.69	16,395 (12.1%)	0.38	119,605 (87.9%)	0.74
Durham Regional Health Unit	640,990	2524	0.50	32,890 (5.1%)	0.48	608,100 (94.9%)	0.51
York Regional Health Unit	1,103,280	1762	0.51	---	---	1,103,280 (100%)	0.52
City of Toronto Health Unit	2,704,415	630	0.54	---	---	2,704,415 (100%)	0.55
Peel Regional Health Unit	1,375,795	1247	0.50	---	---	1,375,795 (100%)	0.50
Wellington-Dufferin-Guelph Health Unit	281,750	4147	0.50	66,025 (23.4%)	0.29	215,725 (76.6%)	0.58
Halton Regional Health Unit	544,030	964	0.64	---	---	544,030 (100%)	0.65

City of Hamilton Health Unit	530,635	1117	0.63	---	---	530,635	0.64
Niagara Regional Area Health Unit	440,970	1854	0.58	14,475 (3.3%)	0.21	426,495 (96.7%)	0.60
Haldimand-Norfolk Health Unit	108,420	2859	0.55	45,150	0.42	63,270	0.66
Brant County Health Unit	132,880	1129	0.53	135 (0.1%)	0	132,745 (99.9%)	0.54
Waterloo Health Unit	529,425	1369	0.57	11,260 (2.1%)	0	518,165 (97.9%)	0.59
Perth District Health Unit	75,624	2219	0.55	44,710 (59.1%)	0.40	30,915 (40.9%)	0.77
Oxford Elgin St. Thomas Health Unit	197,665	2040	0.92	73,945 (37.4%)	0.26	123,720 (62.6%)	0.67
Chatham-Kent Health Unit	101,080	2471	0.52	---	---	101,080 (100%)	0.52
Windsor-Essex County Health Unit	395,105	1851	0.60	20,480 (5.2%)	0.68	374,625 (94.8%)	0.60
Lambton Health Unit	125,215	3002	0.67	29,965 (23.9%)	0.64	95,250 (76.1%)	0.69
Middlesex-London Health Unit	450,130	3317	0.62	16,980 (3.8%)	0.18	433,150 (96.2%)	0.64
Huron County Health Unit	58,360	3399	0.49	58,360 (100%)	0.50	---	---
Grey Bruce Health Unit	159,760	8604	0.57	128,480 (80.4%)	0.53	31,280 (19.6%)	0.70
Simcoe Muskoka District Health Unit	533,720	8800	0.54	161,995 (30.4%)	0.31	371,725 (69.6%)	0.65
Renfrew County and District Health Unit	102,110	15032	0.63	46,415 (45.5%)	0.54	55,695 (54.5%)	0.71
North Bay Parry Sound District Health Unit	121,870	16938	0.51	52,475 (43.1%)	0.39	69,395 (56.9%)	0.61
Timiskaming Health Unit	32,775	14146	0.51	32,775 (100%)	0.52	---	---
Sudbury and District Health Unit	194,050	46551	0.65	31,155 (16.1%)	0.61	162,895 (83.9%)	0.67
Porcupine Health Unit	83,130	271922	0.41	41,650 (50.1%)	0.52	41,480 (49.9%)	0.30

The District of Algoma Health Unit	111,690	41267	0.67	23,955 (21.4%)	0.20	87,735 (78.6%)	0.80
Thunder Bay District Health Unit	139,690	230610	0.66	19,805 (14.2%)	0.63	119,885 (85.8%)	0.75
Northwestern Health Unit	79,085	173828	0.58	64,255 (81.2%)	0.43	14,830 (18.8%)	1.00

Houle SKD, et al. Identifying vaccination deserts: the availability and distribution of pharmacists with authorization to administer injections in Ontario. Can Pharm J (Ott) 2022;155. DOI: 10.1177/17151635221115183.
