# Supplementary material for: Identifying vaccination deserts: The availability and distribution of pharmacists with authorization to administer injections in Ontario
Source: Can Pharm J (Ott). 2022 Aug 5;155(5):258–66. doi: 10.1177/17151635221115183 (PMC9445507; doi:10.1177/17151635221115183)
Supplement: sj-pdf-2-cph-10.1177_17151635221115183 – Supplemental material for Identifying vaccination deserts: The availability and distribution of pharmacists with authorization to administer injections in Ontario [file sj-pdf-2-cph-10.1177_17151635221115183.pdf]

APPENDIX 2 Impact of authorizing all pharmacists to administer injections, by count of Census Subdivisions and public health unit

Injection authorized	No Availability			Below Average Availability		Above Average Availability
**Combination injection authorized and non-authorized**	**Still No Availability n (%)**	**Below Average Availability n (%)**	**Average/Above Average Availability n (%)**	**Below Average Availability n (%)**	**Average/Above Average Availability n (%)**	
The Eastern Ontario Health Unit	1 (6%)			8 (50%)		7 (44%)
	1 (6%)	0	0	6 (38%)	2 (13%)	
City of Ottawa Health Unit	0			0		1 (100%)
	0	0	0	0	0	
Leeds, Grenville and Lanark District Health Unit	7 (32%)			6 (27%)		9 (41%)
	7 (32%)	0	0	5 (23%)	1 (5%)	
Kingston, Frontenac and Lennox and Addington Health Unit	2 (22%)			4 (44%)		3 (33%)
	2 (22%)	0	0	2 (22%)	2 (22%)	
Hastings and Prince Edward Counties Health Unit	10 (56%)			6 (33%)		2 (11%)
	10 (56%)	0	0	3 (17%)	3 (17%)	
Haliburton, Kawartha, Pine Ridge District Health Unit	3 (23%)			5 (38%)		5 (38%)
	3 (23%)	0	0	2 (15%)	3 (23%)	
Peterborough County-City Health Unit	4 (36%)			5 (45%)		2 (18%)
	4 (36%)	0	0	2 (18%)	3 (27%)	
Durham Regional Health Unit	1 (11%)			4 (44%)		4 (44%)
	1 (11%)	0	0	1 (11%)	3 (33%)	
York Regional Health Unit	1 (10%)			5 (50%)		4 (40%)
	1 (10%)	0	0	2 (20%)	3 (30%)	
City of Toronto Health Unit	0			0		1 (100%)
	0	0	0	0	0	
Peel Regional Health Unit	0			2 (67%)		1 (33%)
	0	0	0	1 (33%)	1 (33%)	
Wellington-Dufferin-Guelph Health Unit	6 (40%)			2 (13%)		7 (47%)
	6 (40%)	0	0	2 (13%)	0	
Halton Regional Health Unit	0			1 (25%)		3 (75%)
	0	0	0	0	1 (25%)	

City of Hamilton Health Unit	0			0		1 (100%)
	0	0	0	0	0	
Niagara Regional Area Health Unit	1 (8%)			6 (50%)		5 (42%)
	1 (8%)	0	0	5 (42%)	1 (8%)	
Haldimand-Norfolk Health Unit	0			1 (50%)		1 (50%)
	0	0	0	0	1 (50%)	
Brant County Health Unit	4 (67%)			1 (17%)		1 (17%)
	4 (67%)	0	0	1 (17%)	0	
Waterloo Health Unit	1 (14%)			2 (29%)		4 (57%)
	1 (14%)	0	0	2 (29%)	0	
Perth District Health Unit	1 (17%)			2 (33%)		3 (50%)
	1 (17%)	0	0	2 (33%)	0	
Oxford Elgin St. Thomas Health Unit	3 (19%)			8 (50%)		5 (31%)
	3 (19%)	0	0	7 (44%)	1 (6%)	
Chatham-Kent Health Unit	1 (50%)			0		1 (50%)
	1 (50%)	0	0	0	0	
Windsor-Essex County Health Unit	1 (11%)			2 (22%)		6 (67%)
	1 (11%)	0	0	2 (22%)	0	
Lambton Health Unit	5 (36%)			3 (21%)		6 (43%)
	5 (36%)	0	0	3 (21%)	0	
Middlesex-London Health Unit	5 (42%)			5 (42%)		2 (17%)
	5 (42%)	0	0	5 (42%)	0	
Huron County Health Unit	3 (33%)			2 (22%)		4 (44%)
	2 (22%)	1 (11%)	0	1 (11%)	1 (11%)	
Grey Bruce Health Unit	5 (24%)			7 (33%)		9 (43%)
	5 (24%)	0	0	7 (33%)	0	
Simcoe Muskoka District Health Unit	10 (34%)			10 (34%)		9 (31%)
	6 (21%)	4 (14%)	0	7 (24%)	3 (10%)	
Renfrew County and District Health Unit	9 (43%)			3 (14%)		9 (43%)
	9 (43%)	0	0	1 (5%)	2 (10%)	
North Bay Parry Sound District Health Unit	33 (80%)			3 (7%)		5 (12%)
	31 (76%)	1 (2%)	1 (2%)	0	3 (7%)	
Timiskaming Health Unit	22 (81%)			0		5 (19%)
	22 (81%)	0	0	0	0	
Sudbury and District Health Unit	26 (76%)			0		8 (24%)
	25 (74%)	1 (3%)	0	0	0	
Porcupine Health Unit	21 (70%)			4 (13%)		5 (17%)
	19 (63%)	0	2 (7%)	2 (7%)	2 (7%)	
The District of Algoma Health Unit	24 (80%)			1 (3%)		5 (17%)
	23 (77%)	0	1 (3%)	0	1 (3%)	
Thunder Bay District Health Unit	34 (81%)			2 (5%)		6 (14%)
	34 (81%)	0	0	2 (5%)	0	

Northwestern Health Unit	62 (86%)			5 (7%)		5 (7%)
	61 (85%)	0	1 (1%)	3 (4%)	2 (3%)	

Houle SKD, et al. Identifying vaccination deserts: the availability and distribution of pharmacists with authorization to administer injections in Ontario. Can Pharm J (Ott) 2022;155. DOI: 10.1177/17151635221115183.
